# Systemischer Kompetenzerwerb durch das Transkribieren und Interpretieren psychotherapeutischer Sitzungen

**DOI:** 10.1007/s00729-022-00195-8

**Published:** 2022-04-13

**Authors:** Corina Ahlers, Susanna Kristandl, Kathrin Walch

**Affiliations:** 1 ÖAS, Esslingasse 17/2, 1010 Wien, Österreich; 2 Andreas Lechner Straße 40, 1140 Wien, Österreich

**Keywords:** Transkription, Schlüsselszenen, Textanalyse, Lerneffekte, Therapeutische Performanz, Besondere Momente, „common ground“, Transcription, Key scenes, Text analysis, Learning effects, Therapeutic performance, Special moments of “common ground”

## Abstract

Studierende transkribierten Psychotherapiesitzungen an der Ambulanz der ÖAS. Schlüsselszenen wurden in Interpretationsgruppen und mit der Projektleitung analysiert. Didaktische Effekte zeigen sich in der spezifischen Medialität der Textanalyse und dadurch vielseitigen Konstrukten zur therapeutischen Performanz, oder als Erwerb einer „Fehlerkultur“ und im Erkennen „besonderer Momente“ der therapeutischen Beziehung, v. a. im Vergleich der Kommunikation in Dyaden bzw. in Mehrpersonensystemen.

## Abstract (en)

Students transcribed psychotherapeutic sessions at the ÖAS. Selected key scenes were interpreted in groups and with the project leader. Didactic effects are described, focusing on their awareness through text analysis and elaborating therapeutic constructs, also accepting therapeutic failure and recognizing “key moments” of therapeutic relationships in dyads as compared to those with more participants in therapy.

## Das didaktische Instrument der Textanalyse: Lehr- und Lernziele

Die Analyse von vollständigen Transkripten psychotherapeutischer Sitzungen durch auszubildende und angehende systemische Psychotherapeut*innen erfolgt selten und ist wenig erprobt.^1^ Ausbildner*innen arbeiten heute vor allem mit Videos oder Audios oder in der Anwesenheit von Klient*innen, Studierende und Lehrende verfolgen im Nebenraum über Einwegspiegel oder an Monitoren den Verlauf der Sitzungen. Bücher und Fachartikel bringen Fallvignetten, um die psychotherapeutische Methode am Fall zu explizieren oder theoretische Sichtweisen (Paradigmen) fallempirisch zu unterstützen. Unbeachtet bleiben in den Fallvignetten hingegen Sequenzen, die als unbedeutend oder besonders schwierig gelten. Dies ist insofern nicht einfach hinzunehmen, als es bei den Studierenden den Eindruck erwecken kann, dass psychotherapeutische Sitzungen immer ereignisreich verlaufen und stets kathartische Höhepunkte oder heilende Momente oder Konklusionen hervorbringen, was der langjährigen psychotherapeutischen Erfahrung nicht entspricht. Therapiesitzungen können zuweilen auch scheinbar „ereignislos“ bisweilen sogar als „unangenehm“ empfunden werden. Dass „Ereignisse“ und „Prozesse“ zwar präsent, aber oft verborgen sind und dies mittels einer von Zeitdruck befreiten tiefenhermeneutischen Textanalyse an exemplarischen Fällen gelernt werden kann, ist ein Lehr- und Lernziel der Arbeit an Transkripten.

Ein didaktischer Schritt in diesem Lernprozess ist es, „Schlüsselszenen“ an ihrem metaphorischen Reichtum oder an der Verbalisierung einer signifikanten Veränderung in Wahrnehmung und Erleben von Klient*innen bzw. Therapeut*innen zu erkennen. Dies ist in der von Handlungs- und Zeitdruck befreiten Arbeit am Transkript eher möglich als in der Beobachtung der laufenden psychotherapeutischen Sitzung (Rober 2002, 2010, 2019).

Videoaufnahmen haben in der Entstehungsgeschichte der familientherapeutischen Methode ihren Platz. Bis heute sind Familientherapien von Pionierinnen wie Virginia Satir, Murray Bowen, Nathan Ackermann oder Ivan Boszormenyi-Nagy für Studierende lehrreich. Sie können die Videos u. a. auf Youtube betrachten. Die Teilhabe am systemischen Psychotherapieprozess im Zweikammersystem mit dem Einwegspiegel (Selvini-Palazzoli et al. 1981) hat inzwischen das „Reflektierende Team“ im selben Raum ersetzt (Andersen 1990).^2^ In der Regel ist dies für Klient*innen, die freilich vorab informiert werden müssen, kein Problem. Doch verbietet der Datenschutz die Weitergabe von Videoaufnahmen an die Studierenden der Systemischen Psychotherapie. Die fachspezifische Live Therapie vor den Augen von Studierenden wird seltener. Das Studium der Videos von therapeutischen Sitzungen ist unverzichtbar; doch bleiben die Lerneffekte beschränkt, solange es den angehenden Psychotherapeut*innen an der grundlegenden Kompetenz zur Interpretation der Kommunikation und Interaktion im Mehrpersonen-System fehlt. Genau hierfür mag die tiefenhermeneutische Arbeit an Transkripten eine Grundschulung sein.

Rollenspiele sind ein anderes, in der systemisch-therapeutischen Ausbildung häufig eingesetztes didaktisches Instrument. Corina Ahlers hat die Lehr- und Lernmöglichkeiten im Rollenspiel im Rahmen des systemisch-psychotherapeutischen Curriculums ausführlich untersucht (Ahlers 2017). Ihr Resümee: Das Rollenspiel leide an Verfremdungseffekten, könne zum Kabarett oder Drama mutieren, oder mehr für das Publikum als für die Zwecke der Ausbildung eingesetzt werden. Die Gruppendynamik in der Ausbildungsgruppe spiegele sich im Rollenspiel. Rollenspiele seien daher zwar geeignet, um Techniken des Fragens, Haltungen des neugierigen und aufmerksamen Zuhörens etc. einzuüben. Doch täuschen sie die Studierenden leicht über die Schwierigkeiten im realen Therapieverlauf hinweg, die vor allem aus den dynamischen, stets wirksamen Interessensgegensätzen der Paare, Eltern-Kind-Gruppen etc. resultieren.

Die Arbeit an Transkripten von psychotherapeutischen Sitzungen mit mehr als einer Person (Mehrpersonen-System) stellt demgegenüber eine wichtige didaktische Ergänzung dar, wenn sie nicht sogar basale Bedeutung im Ausbildungsprozess gewinnt. Die vom praktischen Handlungsdruck entlastete Situation der Arbeit am Textprotokoll einer systemisch-therapeutischen Sitzung erlaubt es den Studierenden eher als das Studium von Videos oder das Rollenspiel, die Wahrnehmung der divergenten und oft agonalen Perspektiven der Personen im Mehrpersonen-System zu trainieren und zu professionalisieren, i.e. in das professionelle Selbst des/der systemischen Psychotherapeut*in zu integrieren. Dies und die damit erst mögliche Abstimmung der psychotherapeutischen Interventionen auf das agonale Mehrpersonensystem halten wir für eine Schlüsselqualifikation systemischer Psychotherapeut*innen. Der eingebürgerte Begriff des „common ground“ (Buchholz 2016) wird hier auf das Mehrpersonensystem ausgedehnt, doch ließe sich der Begriff in dieser Hinsicht noch ergänzen: „common but antagonistic ground“.

## Die Pilotstudie zur Analyse von systemisch-therapeutischen Textprotokollen

In den ersten Monaten der Covid-19-Pandemie Anfang 2020 wurde an der Ambulanz der ÖAS ein Forschungs- und Praktikumsprojekt^3^ gestartet, um im ersten Studienabschnitt (erstes und zweites Ausbildungsjahr) in die Praxis und Theorie der systemisch-psychotherapeutischen Arbeit einzuführen und das Interesse der Studierenden an den Interaktionen im Mehrpersonensystem zu wecken. Es wurden genaue, lautgetreue Transkriptionen von systemisch-therapeutischen Sitzungen an der Ambulanz erstellt.

Die Arbeit der Transkription wurde von Studierenden durchgeführt und stellte ihre erste Annäherung an die Sozialkulturalität der Kommunikation dar.^4^ Anschließend wurde in Kleingruppen mit den Transkripten gearbeitet. Die sozial- und kulturwissenschaftlich fundierte Textanalyse wurde im ersten Schritt dazu verwendet, um Schlüsselszenen systemischer Therapieverläufe *textformal* (i.e. an Textmerkmalen der metaphorischen Dichte und/oder der Narration von innerpsychischen oder sozialen bzw. interaktionellen Veränderungen im lebendigen Personensystem) zu identifizieren (Rober 2002, 2010, 2019). Rober orientiert sich am „internal process recall“ (Kagan 1980), wo Therapeut*innen ihre eigene Sitzung interpunktieren und dazu reflektieren. Anders als Kagan geht es ihm um die inneren Dialoge der Studierenden in Bezug auf Text-Sequenzen, welche von diesen als ein „besonderer Moment“ empfunden werden.

Didaktisch angelegt war dieser erste Schritt insofern, als die Studierenden zunächst damit beauftragt wurden, einige Schlüsselszenen vorläufig auszuwählen und sich sodann auf nur zwei Schlüsselszenen zu einigen. Dies spiegelt die Diskursivität im Mehr-Personen-System auf der Ebene der Interpret*innengemeinschaft und macht die Studierenden mit der Strittigkeit und Ambiguität von Sinn und Bedeutung von Aussagen in der sozialen Gruppe vertraut.

Die Wahl von je zwei Schlüsselszenen erfolgte an je verschiedenen Fällen (Transkripten) in 12 Interpretationsgruppen von je sechs Teilnehmer*innen. Die Gruppen einigten sich ohne große Schwierigkeiten auf je zwei Schlüsselszenen. Die Reliabilität scheint im Hinblick auf diese Auswahl hoch. Jedoch ist das Lehr- und Lernziel, jeden Interpretationsvorgang als einen interaktionellen Vorgang zu verstehen, in welchem jeder Beteiligte Bedeutungen zuweist, Emotionen spürt und damit die Voraussetzung für einen dialogischen Verstehensprozess (Anderson und Goolishian 1992) erfüllt, ohnehin wichtiger. Dazu eine exemplarische Aussage einer teilnehmenden Studierenden:

Besondere Momente funkelten mir auch gleich als solche entgegen! Diese Passagen beeindruckten mich persönlich stark, da hatte ich beim Lesen oft Gänsehaut. Diese Beobachtung an mir selbst war dann ein starkes Indiz, neben der inhaltlichen Relevanz der Passage diese als Schlüsselszene zu markieren. Weiters stellte ich mir die Frage, ob ich solche Momente in meinen eigenen Gesprächen auch so klar identifizieren könnte und dass das sehr hilfreich und sinnvoll wäre.

Voraussetzung für die anschließende *thematische* Interpretation der ausgewählten Schlüsselszenen war genau dieses empathische Eintauchen in die Perspektiven der Klient*innen (Anderson und Goolishian 1992). Hier wurden auch Kommunikationsaspekte zwischen Klient*innen und Therapeut*in im Hinblick auf den „common ground“ (Clark 1996; Buchholz 2016) besprochen: Ist der Therapeut/die Therapeutin „allparteilich“? Sind alle Gesprächsteilnehmer*innen noch „im Boot“? Wird ein Klient im Mehrpersonen-System kommunikativ an den Rand gedrängt? Und so fort.

Das Forschungsprojekt entwickelte sich wie in einem anderen Praktikum (Kulturendialog: Ahlers et al. 2020) als Serenpidität, d. h. Forschungsfrage und Vorgangsweisen wurden nicht vollständig vorab bestimmt, sondern während des Projekts mehrfach nach Beobachtungen und Reflexionen verändert (Glück 2020). Die Teilnehmer*innen formulierten am Ende schriftlich Abschlussreflexionen. Dafür gab es aus studientechnischen Gründen zwei mögliche Zeitpunkte: entweder nach Fertigstellung der Transkriptionen (von 1 bis 3 Audios) oder nach der Teilnahme am Interpretationsprozess (Gruppen von 6 Teilnehmer*inne, zwei Transkripte je Person, sechs Gruppensitzungen). Auf diese zwei Zielgruppen an Studierenden bezieht sich die folgende Frage: Was wird gelernt?

Was kann im Prozess des Transkribierens gelernt werden?

Mit Michael Buchholz (2019) sehen wir das psychotherapeutische Gespräch selbst keineswegs als wissenschaftsproduzierten, sondern als „natürlichen“ Gesprächsprozess. Die Verschriftlichung (Transkription) stellt sowohl nach Auffassung der sozialwissenschaftlichen Erzählforschung (Straub 1998) als auch der qualitativen Psychotherapieforschung (Selting et al. 2009) *das Protokoll eines natürlichen Gesprächs *her, und dies macht das natürliche Gespräch in mehreren methodischen Schritten interpretierbar.

Die geforderten Abschlussreflexionen von 31 Transkribierenden wurden qualitativ mit der Methode der qualitativen Inhaltsanalyse (Mayring und Fenzl 2019) nach didaktischen Überlegungen zugunsten der zu erwerbenden psychotherapeutischen Kompetenz kategorisiert.

Ergebnisse der Analyse werden unten tabellarisch vorgestellt.

Tab. 1 zeigt Kategorien von Lernmomenten für die Gruppe A von 31 *Transkribierenden*; Tab. 2 zeigt Kategorien von Lernmomenten für die Gruppe B von 30 *Textintepretierenden;* Tab. 3 zeigt drei Kategorien von Lernmomenten für Gruppe A und Gruppe B.

**Table 1:** 

a. Details in der Kommunikation erkennen	26 RM^5^
b. Imagination des weiteren Verlaufs der Therapie	5 RM

**Table 2:** 

a. Lernen durch Kombination der Literatur und Transkriptanalyse	27 RM
b. Lernerfahrung durch Gruppendiskussion, Austausch mit Lehrtherapeutin	26 RM

**Table 3:** 

Gesamtgruppe 61 Reflexionen	Transkribierend	Interpretierend
a. Beziehung Klient*innen + Therapeut*innen	22 RM‑T^6^	13 RM‑I^7^
b. Praxistransfer: Haltung + Methodenwahl, Gesprächsgestaltung, Technik, Setting, Tempo	25 RM‑T	10 RM‑I
c. Die Identität als Psychotherapeut*in	15 RM‑T	18 RM‑I

### Details der Kommunikation erkennen

Durch das Transkribieren werden bestimmte Gesprächsmomente detailreicher erfasst als im direkten und natürlichen Therapieerlebnis. Gelerntes wird dabei u. a. wie folgt beschrieben:

*Transkription ist natürlich eine sehr, sehr intensive Beschäftigung mit der Klientin und auch mit dem therapeutischen Prozess. Es gibt Stellen, da empfand ich die Klientin z.* *B. stärker ausweichend und bestimmte Themen vermeidend, als mir das beim Beobachten aufgefallen wäre. Manche Dinge kamen sehr viel näher und lösten wiederum eigene Assoziationen aus, wodurch auch wieder neue Hypothesen entstanden*. (RM12)

Das oftmalige Anhören einer Therapiestunde von Anfang bis Ende, das mehrmalige Hören von Sequenzen, um das Gesagte zu verstehen, um die Pausen und die Unterbrechungen einzuschätzen und zu notieren, der Vorgang des Hörens, Wiederhörens und Schreibens, die Diskussion von Sinn und Bedeutung in der Kleingruppe führen die Studierenden in die Medialität des Gesprächs und zugleich in die Agonalität der Kommunikation ein. Derartiges „Durcharbeiten“ kennen wir sonst in der systemischen Ausbildung nicht. Es führt, um das Mindeste zu sagen, zu einem viel genaueren Zuhören Das aktive Zuhören und Niederschreiben ist anders als die Live-Beobachtung, bei der nie so viel Zeit bleibt, die Details zu erfassen.

*Zum einen rückten für mich dadurch die Unterbrechungen im Redefluss der Klientin – Sprechpausen am Ende eines Gedankenganges, das Zögern, das Suchen nach dem richtigen Wort – viel stärker in den Fokus als noch im „Live“– Gespräch oder in der Transkript-Analyse und wurden als taktgebende Elemente des Dialogs sichtbar. Oft nahm hier die Therapeutin den Gesprächsfaden auf bzw. fragte nach. *(RM14)

### Imagination des weiteren Verlaufs der Therapie

Während der Verschriftlichung entwickelten die Studierenden Hypothesen über den weiteren Therapieverlauf. Manche kontaktierten die Forschungsleiterin, um sich nach Fertigstellung des Transkripts zum Fall zu informieren. Die Fragen nach der Zukunft der Therapie reichten von den verdeckten Anliegen der Klienten an die Therapie, mögliche Prognosen, Diagnosen und bisherigen Erfahrungen, welche die Klienten an der Ambulanz gemacht hatten. Der Lerneffekt besteht in der fachspezifischen Kontextualisierung der Therapieereignisse, bzw. das Vorwegnehmen bevorstehender Therapieereignisse durch das genaue Analysieren einer Sitzung.

Was kann in der Interpret*innengruppe gelernt werden?

Hier interpretieren Studierende die Transkripte nach Konzept der Schlüsselszenen (Rober 2002, 2019) als perspektivische Assoziation (Anderson und Goolishian 1992) und als „common ground“ des Kommunikationsgeschehens (Buchholz 2016)^8^ (Tab. 1).

### Lernen durch Kombination von Literatur- und Transkriptanalyse

Lernmomente werden in der Einzelvorbereitung als Markieren von Schlüsselszenen nach dem Literaturstudium genannt. Der Text wird nun anders wahrgenommen, und das „eigene Erleben“ dabei herausgehoben.

RM24: *„Mir hat es gefallen nur anhand eines Textes eine Einheit zu lesen. Das ist ein ganz anderer, neuer Zugang. Erst mit mehrmaligen Lesen gelang es mir, Unterschiede zu bemerken und Bedeutungen zu differenzieren. Der reine Text mit Anmerkungen brauchte besondere Konzentration und eine andere Art von Aufmerksamkeit.“*

RM7 betrachtet es als das „runde Ganze“, trotz Aussparen von Gestik und Mimik!

Zur Schlüsselszene RM4: „Irgendwann wurde mir der Begriff „besonderer Moment“ klarer. Er beschreibt eine positiv konnotierte emotionale Neubetrachtung, die einen Unterschied macht. Es zeigte sich auch, dass er sich im Gesprächsverlauf aufbaut, immer schon mal da ist, wieder untertaucht, neu aufblitzt um irgendwann sein Crescendo zu zeigen, und dann spürt sich etwas anders an. Die Arbeit mit dem rein geschriebenen Wort gibt den Prozessen der Beobachter*innen mehr Raum, eben durch die Uneindeutigkeit und ist auf diesem Weg für das Lernen interessant. Das Lernen darüber, wie man selbst interpretiert, bewertet und empfindet.“

### Lernerfahrung durch Gruppendiskussion und Austausch mit der Lehrtherapeutin

Lernen im Gruppenprozess, in dem die Interpretation von Texten vielseitig und komplex ist. RM26 sieht „common ground“ oder „in einem Boot sein“ oder das „doing to do together“ als relevante Bezeichnungen, um das gute Miteinander im Therapieprozess zu orten. Über die Arbeit in der Gruppe sagt sie: *„Was für mich so schön beim gemeinsamen Arbeiten herauskam ist, dass es so viele unterschiedlichen Wahrnehmungen gibt, wie das Gesagte verstanden werden kann“.*

RM7 kontrastiert unterschiedliches Empfinden verschiedener Gruppenmitglieder bezüglich ein und derselben Sequenz mit der anschließenden Betrachtung der Lehrtherapeutin dahingehend, dass klar wird, wie automatisiert Therapieprozesse erkannt werden.

RM6 fügt dazu: „*Die Begleitung der Lehrenden brachte üppige Dschungeltouren auf den Punkt. Therapeutische Wege durch das Dickicht wurden ausgeschildert und orientierten sich an Zielen und realistischen Möglichkeiten im psychotherapeutischen Prozess. Das routinierte Erkennen der Hauptthemen und die Reduktion auf das Wesentliche waren besonders einprägsam“ (Tab.* 2*).*

### Beziehung Klient*innen + Therapeut*innen

Die Studierenden lernen u. a. die Anpassung der Sprache bzw. Sprechweise zwischen Klient*innen und Therapeut*in. So lernt etwa RM6‑T etwas über den Umgang mit Pausen und Stille, Formulierungen, die im Sprechen verändert werden, Wortwahl, Tempo, auch über inhaltliche Prioritäten- und Themensetzung; über flüssige und stockende Sequenzen, auch über Sackgassen bzw. Fragen der Therapeut*innen, auf die nicht geantwortet wird. Der Einsatz von Füllwörtern („Ähms“ und „Mhms“) sei auf beiden Seiten zu beobachten und indiziere ein kurzes Zögern, eine Suchhaltung oder ein kurzes Nachdenken.

RM5‑T gewinnt eine komplexere Vorstellung von der dialogischen oder multilogischen Gestaltung (Performanz) des psychotherapeutischen Zwei- und Mehrpersonen-Gesprächs, über die unterschiedliche Bedeutung des Wechsels zwischen Sprechen und Pausen, über eine gemeinsame oder eine andere Personen distanzierende Sprache, von der Syntax über Mimik und Gestik bis in die Semantik:

Überraschend war für mich, wie sehr sich die Therapeutin im Laufe der Stunde immer mehr an die Satzstellung der Klientin anpasst. Die Übernahme der Wortwahl nehme ich an, war bewusst, aber die Satzstellung glaub ich war eher unbewusst.

RM2‑I bringt die Flexibilität der Psychotherapeutin gegenüber den Teilnehmer*innen der therapeutischen Sitzung auf den Punkt:

Bei genauerer Betrachtung der therapeutischen Beziehung waren für mich persönlich insbesondere die Gesprächsbeiträge aufschlussreich, die die Lösungsversuche der Klienten würdigen, in denen heikle Themen angesprochen werden und wo eine Konfrontation mit auch schwierigen Themen stattfindet, sowie die starke Orientierung auf die Klient*innen, die in der Gesprächsführung zum Ausdruck kommt.

Auf die Mehrpersonen-Psychotherapie bezogen, sagt RM25-T: *„Besonders beeindruckt hat mich die Intervention, als von einer emotionalen Sprache ins Nonverbale gewechselt wurde und damit eine Veränderung in der Kommunikation bzw. der Begegnung des Paares beobachtbar war“.*

*Ähnlich RM21‑T*: *„Es war spannend zu beobachten, wie das Gespräch zwischen Therapeutin und Klientin eine neue Gestalt annahm und durch die genaue Transkription die Interaktion und das Eingehen aufeinander sichtbar wurde“.*

Erkennbare Unterschiede der therapeutischen Beziehung während der gesamten Therapiesequenz lassen sich für RM24‑I überraschend leicht am Text festmachen und verdichten sich durch das mehrmalige Lesen und durch die ausführliche Diskussion der Lesarten.

### Praxistransfer: Haltung + Methodenwahl, Gesprächsgestaltung, Technik

Lernen als Praxistransfer zeigt sich vor allem im Hinblick auf die im Transkript sichtbar werdende Haltung der Therapeut*innen, im Hinblick auf den in den Gesprächsstrukturen und -Dynamiken erkennbar werdenden Einfluss der bevorzugten Methoden, auf die Technik des Fragens, die Bedeutung der Wahl des Settings und des Tempos der sprechenden Beteiligten, das sie aus oft erkennbaren Gründen der Kommunikations-Sicherung verändern. Dazu gibt RM16‑T folgende Rückmeldung:

Die Klientin spricht immer wieder über eine bestimmte Bubble, in der sie sich befinden würde und die sie offensichtlich als schön empfindet. Die Therapeutin fragt hier mehrfach nach und lässt nicht locker. Ich habe das Gefühl, dass sie wirklich verstehen will, was die Klientin mit dem Begriff Bubble meint.

Das spezifisch passende Verhältnis von therapeutischer Haltung, Setting, Methodenwahl und Gesprächsführung wird für Studierende in der genauen Analyse des Gesprächsverlaufs in seiner Wirksamkeit erkennbar.

Auf die Moderation des Gesprächs gemünzt, sagt RM1-I:

Etwas, was ich mir noch für Klient*innen mitnehme, die sehr viel reden, ist der Tipp, mit Fingerspitzengefühl so zu stoppen (zu unterbrechen), dass ein kongruentes Gespräch entstehen kann, sodass in Folge eine Balance zwischen den Klient*innen und der Therapeut*innen entstehen kann.

Lerneffekte erkennt RM7‑I im Hinblick auf Timing und Kontext der Interventionen:

Wie reagieren die Klienten auf die Fragen der Therapeutinnen und wie gehen diese ihrerseits mit dem Narrativ der Klienten um? Macht es einen Unterschied, wie viel Zeit man den Klienten bei ihrer Antwort lässt? Beziehungsweise wie viel Zeit sollte man generell den Klient*innen geben, um keinen Druck auszuüben und das Gespräch dennoch in Bewegung zu halten?

### Die Identität als Psychotherapeut*in

Die Studierenden reflektieren schließlich auch ihre im Werden begriffene psychotherapeutische Identität durch die Textanalyse.

Für RM1‑I ist es erleichternd zu beobachten, dass „negative Emotionen unweigerlich zum Therapieprozess dazu gehören und nicht Zeichen von unerfahrenen oder schlechten Therapeut*innen sind.“

In der Familientherapie stelle sich, sagt wieder RM1_I, offenbar weniger der Anspruch der Eleganz als vielmehr die Frage der Wirkung (Effizienz). Dass der therapeutische Effekt in der Einzeltherapie eher als im Mehrpersonensystem „fühlbar“ werde, weil die Parteilichkeit nicht gleichmäßig verteilt werden muss, habe (RM1-I) in der Arbeit mit Transkripten von Einzeltherapien wahrgenommen.

RM25‑I ordnet ihre Gefühle zur psychotherapeutischen Identität, indem sie durch das Bearbeiten der Transkripte die Therapeut*innen-Rolle entmystifizieren will und die Wichtigkeit der Selbstfürsorge erkennt. Einen interessanten Holperer erzählt RM4‑T. Sie erschreckt ihre Töchter, weil sie mit den Kopfhörern transkribiert und plötzlich laut ausruft*: „Jetzt lasst’s ihn halt mal ausreden!“* Das Transkribieren helfe ihr, die Herausforderung der Allparteilichkeit in der Paartherapie zu erleben und die eigenen Gefühle zu analysieren und zu merken, wie wichtig die Position der Multiperspektivität im Mehrpersonensystem ist.

RM13‑T beschreibt die Wirkung der Kopfhörer während des Transkribierens als erlebnisintensivierend. Sie stellt sich Fragen wie *„um was geht’s der Klientin?“* und *„was hat das Thema mit mir zu tun bzw. was löst es bei mir aus?“*

Die virtuelle Übernahme der Handlungsperspektive der Therapeut*in wird von mehreren Studierenden als lehrreich empfunden. Sie stellen Überlegungen an, *„wie man selber in diesen konkreten Fällen interveniert hätte“ *(RM17-I). RM24‑T beobachtet das abgehackte Sprechen, das Nicht-Beenden von Sätzen und macht sich zum Vorsatz, in der therapeutischen Rolle solches zu vermeiden: *„Es ist mir aufgefallen, dass die Klientin teilweise nicht in ganzen Sätzen redet. Daher habe ich mir mitgenommen, auf meine Sprechgeschwindigkeit zu achten und merke jetzt selbst, wenn ich nicht in ganzen Sätzen spreche. Es ist mir positiv aufgefallen, dass die Therapeutin die Klientin ausreden lässt, Sprechpausen nutzt und mit der Sprachgeschwindigkeit entschleunigend wirkt. Das möchte ich mir für mein Arbeiten auch mitnehmen.“*

## Relevanz der Forschung für die Ausbildung von systemischen Psychotherapeut*innen

Das vorgestellte Forschungspraktikum bestimmt seine Praxisrelevanz aus Erkenntnissen für eine weiter auszuarbeitenden Didaktik der Ausbildung in systemischer Paar‑, Gruppen- und Einzelpsychotherapie. Aus unserer Sicht wird Praxisrelevanz der Arbeit an Transkripten durch folgende Lernprozesse erreicht:

1. Die Findung und Analyse von Schlüsselszenen einer Therapiestunde schult die Wahrnehmung des inneren therapeutischen Dialogs. Sie schult die Reflexion dieses inneren Dialogs und bezieht die Person des Therapeuten/der Therapeutin in das Konstrukt von der jeweiligen therapeutischen Performanz ein.
2. Durch die Übung und Praxis in Textanalyse (die in der Regel aus stets schon interpretierender Transkription, formaler und thematischer Textanalyse besteht) entwickeln Studierende ein Gefühl für die spezifische Medialität des psychotherapeutischen Gesprächs: Sie achten genauer auf Zwischentöne, Sprachmelodie, Abbruch oder Schweigen. Sie lernen schon durch den Akt der Transkription die Momente der sprachlichen Performanz: Semantik und Syntax, direkte und indirekte Rede, biographische Kommentare, Evaluationen, die Grundstruktur der Geschichte (story), die „Sparkling Moments“ (Freedman und Combs 1996), in denen eine Interaktion emotional aufgeladen und „knisternd“ wird, Momente, die eher „gefühlt“ als kognitiv verstanden werden. Die Textanalyse eines Textprotokolls ist in diesen Hinsichten weitaus präziser und somit lehrreicher als die direkte Beobachtung einer Psychotherapie, welche viele Details der psychotherapeutischen Interaktion in ihrer Wirkung und Bedeutung kaum fassen kann, weil ein Vorgang allzu schnell vorüberzieht und ein nächster schon passiert.
3. Wirkungen oder Effekte psychotherapeutischer Interventionen im Therapieprozess werden durch die Arbeit an Transkripten exemplarisch gefasst und reflektierbar gemacht. Anders gesagt: die beschriebene Arbeit an Transkripten wird zur psychotherapeutischen Werkstatt (zu einer von mehreren möglichen), in der die angehenden ebenso wie die lehrenden Psychotherapeut*innen als therapeutisch interagierende Personen mit erfasst und zum Gegenstand von solidarischer Kritik und Reflexion werden.
4. Studierende lernen in der Textprotokolle analysierenden Arbeitsgruppe unterschiedliche Lesarten, Positionen, Haltungen und Ansätze kennen. Dies vermehrt die Fähigkeit zu hypothetischem Denken. Diese Fähigkeit wird durch die Bearbeitung des konkreten Textes unter Einbeziehung aller handelnden Personen und ihren zum Teil widersprüchlichen Auffassungen, Meinungen und Interessen gefördert. Diese Lehr- und Lernarbeit hat daher eine andere didaktische Wirkung als etwa kurze Vor- und Nachgespräche zum Erleben einer psychotherapeutischen Sitzung. Die Fehlerkultur wird gepflegt, die Ansprüche an die Therapeuten-Rolle hinterfragt. Realistischere Bilder zur psychotherapeutischen Kompetenz können entstehen. Selbstüberforderung und Ängste dürften damit gemildert werden.

## Footnotes

1 In der SFU/ÖAS Kooperation bei Dr. in Gerda Mehta, in der ÖAS/Wien bei Univ.-Doz.in Dr.in Corina Ahlers.

2 Das Reflektierende Team von Tom Andersen (1990) besteht aus der professionellen Reflexion vor den Klient*innen. Die Klient*innen werden dann, darauf bezugnehmend, befragt.

3 Das Projekt wurde von Univ.-Doz.in Dr.in Corina Ahlers konzipiert, verwaltet und didaktisch begleitet.

4 Corina Ahlers adaptierte ein leicht durchführbares Manual für das Transkribieren im Sinne der Konversationsanalyse (GAT) nach Selting et al. (acc.am 30.10.2021).

5 RM steht für Rückmeldungen der Teilnehmer*innen.

6 RM‑T für Rückmeldung Transkribierend.

7 RM‑I für Rückmeldung Interpretierend.

8 Sichtweisen zum Text erweitern sich, indem an einer Sequenz vielseitige Bedeutungen generiert werden, die verglichen und auf ihre Plausibilität geprüft werden. Die Gruppe findet Alternativen, die im nächsten Schritt mit der Lehrenden kontrastiert werden.

## References

[CR1] Ahlers C (2017). Kommunikative Kompetenz. Das Rollenspiel in der systemischen Psychotherapie.

[CR2] Ahlers C, Vittorelli N, Glück G, Nakshbandi A (2020). Die Anderen sind wir. Eine Anleitung zum Umgang mit kultureller Uneindeutigkeit.

[CR3] Andersen T (1990). Das reflektierende Team: Dialoge und Dialoge über Dialoge.

[CR4] Anderson H, Goolishian H (1992). Der Klient ist Experte: Ein therapeutischer Ansatz des Nicht-Wissens. Z system Ther.

[CR6] Buchholz MB (2016). Conversational errors and common ground activities in psychotherapy—insights from conversation analysis. International Journal of Psychological Studies.

[CR5] Buchholz MB (2019). Szenisches Verstehen und Konversationsanalyse. Psyche.

[CR7] Clark HH (1996). Using language.

[CR8] Freedman J, Combs G (1996). Narrative therapy: the social construction of preferred realities.

[CR9] Glück G, Ahlers C, Vittorelli N, Glück G, Nakshbandi A (2020). Das Projekt Kulturendialog. Die Anderen sind wir. Eine Anleitung zum Umgang mit kultureller Uneindeutigkeit.

[CR10] Kagan N, Hess AK (1980). Influencing human interaction: eighteen years with IPR. Psychotherapysupervision: theory, research, and practice.

[CR11] Mayring P, Fenzl T, Baur N, Blasius J (2019). Qualitative Inhaltsanalyse. Handbuch Methoden der empirischen Sozialforschung.

[CR12] Rober P (2002). Constructive hypothesizing, dialogic understanding and the therapist’s inner conversation: some ideas about knowing and not in the family therapy session. Journal of Marital and Family Therapy.

[CR13] Rober P (2010). Die innere Konversation des Familientherapeuten. Über das Selbst des Therapeuten, therapeutische Sackgassen und wie man darüber nachdenken kann. Familiendynamik.

[CR14] Rober P (2019). Die Komplexität des Zuhörens. Kontext.

[CR15] Selting, M., Auer, P., Barden, B., Bergmann, J., Couper-Kuhlen, E., Günthner, S., Meier, Ch , Quasthoff, U., Schlobinski, P., & Uhmann, S. (2009). Gesprächsanalytisches Transkriptionssystem (GAT). https://www.researchgate.net/publication/292769316_Gesprachsanalytisches_Transkriptionssystem_GAT_2. Zugegriffen: 30. Okt. 2021.

[CR16] Selvini-Palazzoli M, Boscolo L, Cecchin G, Prata G (1981). Hypothetisieren – Zirkularität – Neutralität. Drei Richtlinien für den Leiter einer Sitzung. Familiendynamik.

[CR17] Straub J, Straub J (1998). Geschichten erzählen, Geschichte bilden. Grundzüge einer narrativen Psychologie historischer Sinnbildung. Erzählung. Identität und historisches Bewusstsein. Die psychologische Konstruktion von Zeit und Geschichte. Erinnerung, Geschichte, Identität I.

